# Perceptions of burden of caregiving by informal caregivers of cancer patients attending University of Calabar Teaching Hospital, Calabar, Nigeria

**DOI:** 10.11604/pamj.2014.18.159.2995

**Published:** 2014-06-18

**Authors:** Paulina Ackley Akpan-Idiok, Agnes Nonye Anarado

**Affiliations:** 1 Department of Community Health Officers’ Training Programme, University of Calabar Teaching Hospital, Calabar, Nigeria; 2 Department of Nursing Sciences, Faculty of Health Sciences and Technology, University of Nigeria, Enugu, Nigeria

**Keywords:** Perceptions of burden, informal caregivers, cancer patients, caregiving, Nigeria

## Abstract

**Introduction:**

Cancer care is devastating to families. This research studied the informal caregivers’ perceptions of burden of caregiving to cancer patients attending University of Calabar Teaching Hospital, Calabar.

**Methods:**

The research adopted a cross-sectioned descriptive design and 210 caregivers providing care to advanced cancer patients were purposively selected. Data were collected using a researcher developed questionnaire and standardized Zarit Burden Interview scale (ZBIS). Data collected were analysed using descriptive and chi-square statistics with the help of SPSS 18.0 and PAS 19.0 softwares.

**Results:**

The results indicated that the caregivers were in their youthful and active economic age, dominated by females, Christians, spouses, partners and parents. The burden levels experienced by the caregivers were as follows: severe (46.2%), moderate (36.2%) and trivial of no burden (17.6%). The forms of burden experienced were physical (43.4%), psychological (43.3%), financial (41.1%) and social (46.7%), quite frequently and nearly always. Psychological and social forms of burden had the highest weighted score of 228 in terms of magnitude of burden. The result further showed that there was a significant (P = 0.001) and inverse association between caregivers’ burden and the care receivers’ functional ability. The level of burden also increased significantly (P = 0.000) with the duration of care, while there was also a significant (P = 0.01) relationship between caregivers’ experience of burden and their desire to continue caregiving.

**Conclusion:**

Caregiving role can be enhanced by provision of interventions such as formal education programme on cancer caregiving, oncology, home services along side with transmural care.

## Introduction

Cancer is one of the leading causes of adult deaths and it accounted for 7.6 million deaths worldwide in 2008. Statistics have shown that the disease killed over 23,681 and 23,775 persons in Turkey in 2000 and 2003 as well as 635,000 and 556,400 persons in India in 2008 and 2010 respectively. In the United States of America (USA) and Africa, 559,650, 542,000 persons died of cancer in 2007. In Nigeria, death toll of 53,064 persons was reported in 2008 [1–3]. The recent new cases of cancer diagnosed were 1.5 million in America in 2009; 715,000 cases in Africa in 2008; and 500,000 cases in Nigeria in 2010. The projected new cases of cancer for the world will stand at 27 million, while that of Africa will stand at 1.52 million by 2030 [1, 3, 4]. The rising figure may become a major challenge for caregiving with its attendant burden on the caregiver.

Caregivers’ perceptions of burden refer to various ways informal caregivers of advanced cancer patients describe and rank their caregiving experience as challenging, while burden refers to the feelings, needs, difficulties, pains and all forms of distress experienced by caregivers of cancer patients. Caregivers’ burden therefore is considered “a multidimensional bio-psychosocial reaction” resulting from an imbalance of care demands, relative to caregivers’ personal time, social, roles, physical and emotional states, financial resources and formal care recourses given the other multiple roles they fulfill [2, 5–7].

Studies have shown that informal caregivers, typically family members or friends provide care to advanced cancer patients. They play an essential role, usually unpaid, in caring for patients with cancer. Most advanced cancer patients have diverse needs which include assistance with medication, transportation for treatment, activities of daily living and emotional support which include helping patients with their self-care, managing their treatment and symptoms as well as with the suffering of a family member [8]. The burden experienced by them is commonly perceived as chronic stressors and they often experience negative psychological, behavioural, economic and physiological effects on their daily lives and health [9, 10]. They are also affected by other stressors such as changes in roles and employment and disruptions in schedules (e.g. frequent clinic visits). Caregivers may suffer social and economic deficits such as lifestyle disruption, less socializing and greater out-of-pocket and lost productivity cost [2].

There is dearth of information highlighting the caregivers’ perceived burden of caregiving to cancer patients in University of Calabar Teaching Hospital, Calabar, Nigeria. Therefore, the research objectives were to: (i) describe the socio-economic characteristics of caregivers, (ii) determine their perceived levels of burden of caregiving to advanced cancer patients, (iii) examine the various forms of burden experienced by caregivers, (vi) determine the functional ability of the care receiver and relate them to their perceived burden, (v) determine the relationship of duration of care to caregivers’ perceived burden, (vi) relationship between perceived burden and desire to continue caregiving.

## Methods

### Study Area/study population

Cross-sectional descriptive survey design was carried out among eligible 210 cancer caregivers attending University of Calabar Teaching Hospital in Calabar Metropolis, Cross River State, Nigeria. Calabar is located at the extreme of Southern Senatorial District of Cross River State. The geographical location of Calabar urban is latitude 4°58’ North and 8°17 East. It has a common boundary with the republic of Equatorial Guinea to the South, in the West, Oron Local Government Area of Akwa Ibom State, in the East, Akpabuyo Local Government Area in Cross River State and bounded by Odukpani Local Government Area in the North. This hospital serves as referral centre to other hospitals, health centres and other health institutions where cancer patients are admitted. Apart from being a referral centre, it is an embodiment of all cultural groups within and outside Nigeria. The population includes all advanced (stages iii and iv) cancer patients in the study area. The participants for the study would cut across all ethnic groups and diversified culture from within and outside the State.

### Ethical Considerations

The principles and guidelines for international bioethical standard were adhered to throughout the study. Permission to undertake this research was obtained from the Head of the Ethical Committee of the hospital and informed consent gained from each informal cancer caregiver. The purpose of the research was explained to the caregivers and were assured of their right to withdraw from the study at any stage.

### Sample and instrument

Using “a priory computer power analysis software (G Power 3.1.5) calculator” [11], 210 respondents were purposively sampled for the study. This sampling method allowed for wider coverage of the study respondents. A validated questionnaire divided into three sections namely: socio-economic characteristics, duration of care and functional levels of care receiver were developed by the researcher. Also adopted was 22 item standardized zarit burden interview (ZBI) 5 - point likert scale (never = 0, rarely = 1, sometimes = 2, quite frequently = 3 and nearly always = 4) for measuring caregivers’ burden. The sum of burden was achieved by adding the scores for all items with a range of 0 - 88 with higher scores indicating severity (higher levels) of burden [12]. The Zarit Burden Interview (ZBI) instrument was subjected to test-retest reliability and construct validity; the reliability coefficient of the instrument was 0.994 and has been used in some studies conducted in Nigeria [13, 14].

### Method of data analysis

Analysis of data was carried out using the computer software programme Statistical Package for the Social Sciences (SPSS) version 18.0, Predictive Analytical Software (PAS), version 19.0. Descriptive statistics (means, standard deviation and percentages) and chi-square analysis were used to analyze the data.

## Results

### Socio-economic characteristics of caregivers

The study indicated that informal caregivers were in their youthful and active economic age (< 31 - 50 age bracket, mean age: 35.9 ± 18.1), mostly Christians, 168 (80.0%) dominated by females 132 (62.9%), spouses, partners43 (20.5%) and parents 132 (62.9%), 81 (38.6%) were unemployed, 114 (58.8%) had secondary and tertiary education (Table 1).

**Table 1:** Socio-demographic characteristics of informal care givers

Characteristics	Frequency	Percentage
**Gender**		
Male	78	37.1
Female	132	62.9
**Age**		
< 30 years	79	37.6
31-50 years	97	46.2
51 -70 years	34	16.2
Mean	35.9 (SD ± 18.1)	
**Religion**		
Christianity	168	80.0
Muslim	18	8.6
Others	24	11.4
**Marital Status**		
Married	98	46.7
Single	57	27.1
Divorced	12	5.7
Widowed	43	20.5
**Educational qualification**		
No Formal education	21	10.0
Primary	74	35.2
Secondary	83	39.6
Tertiary	32	15.2
**Employment/Work Status**		
Not employed	81	38.6
Artisans	10	4.8
Traders	21	10
Farmers	15	7.1
Contractors	4	1.9
Retiree	50	23.8
Civil/Public Servants	19	9
Student/Apprentice	10	4.8
**Relationship to care receiver**		
Parent	132	62.9
Spouse/partner	43	20.5
Sibling	21	10
Friend	10	4.8
Brethren	4	1.9

### Perceived levels of burden of caregiving to advanced cancer patients

**Figure 1:**
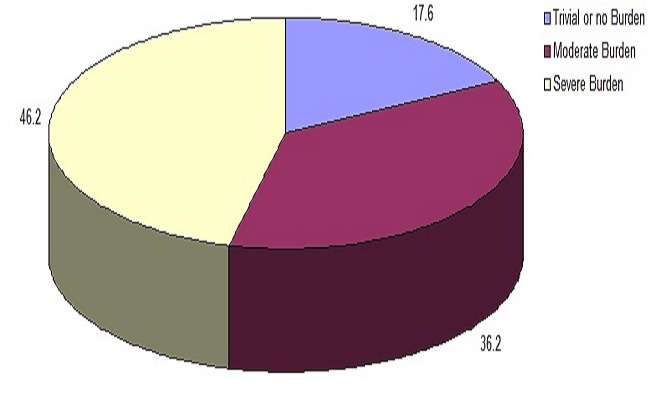
Distribution of perceived levels of burden of caregiving to advanced cancer patient

The caregivers under study experienced trivial or no burden 37 (17.6%), moderate 76 (36.2%) and severe burden 97 (46.2%) (Figure 1).

### Forms of burden experienced by caregivers of advanced cancer patients

The informal caregivers in the study experienced the following forms of burdens: physical (91 (43.3%), psychological 91 (43.3%), financial 87 (41.4%) and social 98 (46.7%), with psychological and social forms of burden having the highest magnitude of burden (weighted score of 228; Table 2).

**Table 2:** Distribution of forms and magnitude of burden experienced by cancer caregivers n = 210

				Forms and magnitude of Burden					
Occurrence		Physical Frequency (%)	Weighted score	Psychological Frequency (%)	Weighted score	Financial Frequency (%)	Weighted score	Social Frequency (%)	Weighted score
**Never**	**0**	36 (17.1%)	0	35 (16.6%)	0	31 (14.8%)	0	40 (19.1%)	0
**Rarely**	**1**	38 (18.1%)	38	39 (18.6%)	39	35 (16.6%)	35	28 (13.3%)	28
**Sometimes**	**2**	45 (21.4%)	90	45 (21.4%)	90	57 (27.2%)	114	44 (21.0%)	88
**Quite frequently**	**3**	40 (19.1%)	120	34 (16.2%)	102	41 (19.5%)	123	41 (19.5%)	123
**Nearly always**	**4**	51 (24.3%)	204	57 (27.1%)	228	46 (21.9%)	184	57 (27.1%)	228
**Total**	**10**	210 (100)	210 (100)	210 (100)	210	210 (100)	210	210	210
Range		45.2		45.9		45.6		46.7	

### Functional abilities of care receivers on caregivers’ burden

Considering functional ability variables of care receivers (Table 3) where a scoring percentage of 50 and above for “yes” indicated low functional ability, the care receiver (cancer patient) depended on the caregivers in performing daily living activities except in grooming (11.0%), taking medication (5.7%), using telephone (15.2%) and wandering (19.1%).

**Table 3:** Functional status of care receivers

S/N	Functional ability of care receiver variables	No	%	Yes	%
**A**	Eating (needs someone to feed him or her)	54	25.7	156	74.3
**B**	Bathing/showering	48	22.9	162	77.1
**C**	Dressing (choosing) put on appropriate clothing	53	25.2	157	74.8
**D**	Grooming (brushing hair, teeth)	187	89.1	23	11.0
**E**	Using the toilet	59	28.1	151	71.9
**F**	Incontinence	38	18.1	172	81.9
**G**	Transferring from bed/chair/car	21	10.0	189	90.0
**H**	Preparing meals	28	13.3	182	86.7
**I**	Staying alone, must be supervised	14	6.7	196	93.3
**J**	Taking medication	198	94.3	12	5.7
**K**	Managing money or finance	7	3.3	203	96.7
**L**	Performing household chores	47	22.4	163	77.6
**M**	Using the telephone	178	84.7	32	15.2
**N**	Mobility	68	32.4	142	67.6
**O**	Wandering, or the potential to wander	170	80.0	40	19.1

NB: A score of 50% and above for (yes) indicates low functional ability

### Relationships between functional ability of care receivers (cancer patients) and burden levels of caregivers

The study revealed that majority, 137 (65.2%) of the care receivers had low functional ability, while 73 (35.8%) had high functional ability (Table 4). On burden level, 71 (51.8%) of caregivers experienced severe burden level, while 27 (37.1%) caregivers who provided care to care receivers with high functional ability reported trivial or no burden.

**Table 4:** Relationship between functional ability of care receivers and burden level of cancer caregivers. n = 210

Burden level	Functional ability of care receivers		N	P value ≤
	Low (%)	High (%)		
**Trivial or no burden**	10 (7.3)	27 (37.1)	37	.001
**Moderate burden**	56 (40.9)	20 (27.4)	76	.001
**Severe**	71 (51.8)	26 (35.6)	97	.001
**Total**	137 (65.2%)	73 (34.8%)	210	.001

NB: Significant (P ≤ .001) inverse relationship between the caregivers’ burden and functional ability

Also, the chi-square statistical test of relationships proved that there was a significant (P = 0.001) inverse relationship between the caregivers’ burden and the care receivers’ functional ability.

### Forms of burden of caregivers and functional ability of care receivers (cancer patients)

The caregivers of cancer patients with low functional ability reported physical burden of 46.7%, financial burden of 25.6%, social burden of 17.5% and low psychological burden of 10.2% (Table 5). Relatively, psychological burden (31.5%), physical burden (24.6%), financial burden (20.6%) and social burden (22.3%) were reported by caregivers of cancer patients with high functional ability (Table 5).

**Table 5:** Forms of burden of caregivers and functional ability of care receivers (n = 210)

Functional ability	Forms of burden				
	Physical Frequency (%)	Psychological Frequency (%)	Financial Frequency (%)	Social Frequency (%)	Total	P value ≤
**Low**	64 (46.7)	14 (10.2)	35 (25,6)	24 (17.5)	137	.001
**High**	18 (24.6)	23 (31.6)	15 (20.6)	17 (23.3)	73	

### Relationship between duration of care and perceived levels of burden of caregivers of cancer patients

Considering duration of care and burden level (Table 6), majority 20(54.1%) of the caregivers, under 1 - 5 months bracket experienced trivial or no level of burden; 30 (39.5%) of caregivers under 6 - 10 months bracket had moderate burden, while 55 (56.7%) of caregivers at 11 months or above suffered severe burden. Also, there was a statistical significant relationship, (P < = 0.001) that existed between duration of care and caregivers’ burden.

**Table 6:** Relationship between levels of burden of caregivers of cancer patients and duration of care n = 210

	Burden level			P value ≤
Duration of care (months)	Trivial or no burden Frequency (%)	Moderate burden Frequency (%)	Severe burden Frequency (%)	
**1 – 5**	20 (54.1%)	10 (13.2%)	14 (14.4%)	0.001
**6 – 10**	10 (32%)	30 (39.5%)	28 (28.9%)	
**11 and above**	7 (18.9%)	36 (47.4%)	55 (56.7%)	
**Total**	37	76	97	

### Relationship between perceived burden and desire to continue caregiving

The study revealed that majority (58.6%) respondents desired to continue caregiving despite their perceived burden levels of trivial or no burden (21.7%), moderate (14.58%) and severe burden (56.8%), while 41.4% of the respondents desired to opt out probably due to fears and uncertainty of the disease prognosis (Table 7). There was a statistical significant (P = 0.01) relationship between caregivers’ experience of burden and their desire to continue caregiving.

**Table 7:** Perceived burden and desire to continue care giving

Desire to continue caregiving role	Burden levels			Total%	df = 2	X^2^ cal.	X^2^ tab
	Trivial or no burden	Moderate burden	Severe burden				
**Not to continue (6 – 12)**	7 (15.3)	16 (31.5)	64 (40.2)	87	2	44.76	5.99
**To continue (13 – 24)**	30 (21.7)	60 (44.5)	33 (56.8)	123			
**Total**	37	76	97	210			

## Discussion

Demographic characteristics of the respondents and perceived issues are the crucial factors that can influence informal cancer caregivers. Although caregiving cuts across religions, majority of the respondents were Christians. This is expected given that the study area is predominantly Christian. Similar results have been reported by previous researchers [2, 5, 7, 9, 15].

Levels of burden in this study refer to the ranking of the impacts of caregiving responsibility on the caregiver's life. Applying the four point Zarit Burden Scale of trivial or no burden (0 - 20), mild (21 - 30), moderate (31 - 40) and severe burden (41 - 88), The findings are consistent with the works of [16, 17] who reported trivial or no burden, moderate and severe burden levels in Eastern United States of America. However, other studies with low level of burden (15%) experienced by caregivers were ascribed to available forms of materials and human interventions such as rapport, counselling, health education, social and financial provisions to cancer caregivers [7, 18, 19].

Caregivers are often faced with concurrent stressful events and extended, unrelenting stress, so, forms of burden include physical, psychological, social and financial [2, 10, 20]. These findings are consistent with the previous studies that reported extremely high level of physical, psychological, social and financial burden in caregiving to advanced adult cancer patients [2, 9, 10, 21].

Functional ability is defined as the enormous physical and existential challenges encountered by cancer patients in performing the activities of daily living (ADL) [20]. Caring for patients with comorbidities and cancer related impairments is excruciatingly challenging. The study highlighted that the informal caregivers to advanced cancer patients in the study location experienced severe burden. Researchers reported that burden experienced by caregivers was related to functional levels of care receivers (cancer patients) [1, 19, 20, 22]. Studies also proved that helping patients with personal tasks such as washing and eating appeared to be more stressful to caregivers than helping with non personal tasks such as grocery shopping [23].

The functional ability of care receivers relates to the burden levels of informal caregivers [7, 24]. This implies that the higher the care receiver functional ability, the lower the level of burden experienced by the caregivers. The cohorts of current study have advanced cancer patients with low ability for activities of daily living, indicating that the informal caregivers played a central role in managing all aspects of the patients’ care in the study location. The findings upheld the works of [25] and [26] who had reported low functional ability for care receivers and severe burden for informal caregiviers.

Burden of care refers to the extent of work load experienced on caregivers’ life and includes physical, psychological, financial and social among others [27, 28]. The study highlighted that, caregiving to a cancer patient was burdensome, but the magnitude of the burden depended on the functional ability of the care recipient; where the functional abilities of the care receivers were low or high, the caregiver experienced a variety of burden such as physical, financial, psychological and social. This is an indication that the informal caregivers developed resilience to all forms of caregiving burden [2, 10, 29–32]

In this study, duration of care refers to amount of time in months devoted by informal caregivers to cancer patients. This is an indication that the more years/months/hours a person spends with caregiving, the more the burden of care increases. Studies have shown that caregivers take away time that could be spent in paid employment and that duration of care in hours/months determines the extent of caregivers’ burden [19, 33]. According to [10], caregiving is labour intensive, with approximately one-quarter of those caring for cancer patients spending in excess of 40 hours a week providing services to family or friends living with cancer.

Caregivers’ experience of burden and their desire to continue caregiving is an indication that burden perceptions influences caregivers’ desire to continue cancer caregiving. The findings suggest that informal caregivers’ desire to continue might have arisen from their inherent resilience, optimistic personality, traditional family structure/cultural bonds of the African society and probably from adoption of problem-solving coping strategies. Similar findings were reported among caregivers of cancer and epilepsy patients in Nigeria [14, 34].

### Limitations

This study is a cross-sectional study conducted in just one Teaching Hospital in the country. The researcher also excluded formal caregivers of cancer patients in the study location. Nevertheless, this study was conducted to investigate only burden and benefits of cancer caregiving without including support and intervention strategies for the caregivers.

## Conclusion

The informal caregivers of cancer patients experienced severe burden, physical, psychological and social forms of burden in University of Calabar Teaching Hospital, Calabar, Nigeria. Generally, caregiving by informal cancer caregivers was highly burdensome because care receivers had low ability for activities of daily living. The burden of care increased with the length of time (months) spent in caregiving. The informal caregivers desired to continue role despite their perceived burden. It is also expedient to embark on early assessment, nursing diagnosis and potential interventions to reduce cancer informal caregivers’ burden. Caregiving role can be enhanced by provision of interventions such as formal education programme on cancer caregiving, oncology home services alongside with transmural care consisting of communication and continuity of care.
